# Antagonistic Regulation of Parvalbumin Expression and Mitochondrial Calcium Handling Capacity in Renal Epithelial Cells

**DOI:** 10.1371/journal.pone.0142005

**Published:** 2015-11-05

**Authors:** Thomas Henzi, Beat Schwaller

**Affiliations:** Anatomy, Department of Medicine, University of Fribourg, Fribourg, Switzerland; Cinvestav-IPN, MEXICO

## Abstract

Parvalbumin (PV) is a cytosolic Ca^2+^-binding protein acting as a slow-onset Ca^2+^ buffer modulating the shape of Ca^2+^ transients in fast-twitch muscles and a subpopulation of neurons. PV is also expressed in non-excitable cells including distal convoluted tubule (DCT) cells of the kidney, where it might act as an intracellular Ca^2+^ shuttle facilitating transcellular Ca^2+^ resorption. In excitable cells, upregulation of mitochondria in “PV-ergic” cells in PV-/- mice appears to be a general hallmark, evidenced in fast-twitch muscles and cerebellar Purkinje cells. Using Gene Chip Arrays and qRT-PCR, we identified differentially expressed genes in the DCT of PV-/- mice. With a focus on genes implicated in mitochondrial Ca^2+^ transport and membrane potential, uncoupling protein 2 (*Ucp2*), mitocalcin (*Efhd1*), mitochondrial calcium uptake 1 (*Micu1*), mitochondrial calcium uniporter (*Mcu*), mitochondrial calcium uniporter regulator 1 (*Mcur1*), cytochrome c oxidase subunit 1 (*COX1*), and ATP synthase subunit β (*Atp5b*) were found to be up-upregulated. At the protein level, COX1 was increased by 31 ± 7%, while ATP-synthase subunit β was unchanged. This suggested that these mitochondria were better suited to uphold the electrochemical potential across the mitochondrial membrane, necessary for mitochondrial Ca^2+^ uptake. Ectopic expression of PV in PV-negative Madin-Darby canine kidney (MDCK) cells decreased COX1 and concomitantly mitochondrial volume, while ATP synthase subunit β levels remained unaffected. Suppression of PV by shRNA in PV-expressing MDCK cells led subsequently to an increase in COX1 expression. The collapsing of the mitochondrial membrane potential by the uncoupler CCCP occurred at lower concentrations in PV-expressing MDCK cells than in control cells. In support, a reduction of the relative mitochondrial mass was observed in PV-expressing MDCK cells. Deregulation of the cytoplasmic Ca^2+^ buffer PV in kidney cells was counterbalanced *in vivo* and *in vitro* by adjusting the relative mitochondrial volume and modifying the mitochondrial protein composition conceivably to increase their Ca^2+^-buffering/sequestration capacity.

## Introduction

Parvalbumin (PV) is a cytosolic protein of the family of EF-hand Ca^2+^-binding proteins. It is commonly considered as an intracellular Ca^2+^ buffer or more precisely a Ca^2+^ signal modulator due to its two high-affinity Ca^2+^/Mg^2+^ mixed sites. PV is expressed at high levels in neuron subtypes, e.g. in certain GABAergic interneurons, in fast-twitch muscles, in parathyroid glands and in some epithelial cells in the kidney [1,2]. The physiological role of PV has been most thoroughly investigated in fast-twitch muscles and the brain, where it mainly acts as a slow-onset Ca^2+^ buffer modulating the temporal and spatial aspects of Ca^2+^ transients. PV-deficient (PV-/-) mice develop and breed normally and no obvious alterations in their home cage behavior or physical activity are observed [3]. While the prolonged contraction-relaxation cycle of fast-twitch muscles observed in *tibialis anterior* (TA) from PV-/- mice was as expected from the absence of a slow-onset Ca^2+^ buffer, the enhanced resistance to muscle fatigue came as a surprise and was shown to be the result of an upregulation of mitochondria. The relative mitochondrial volume in fast-twitch muscles, e.g. in *extensor digitorum longus* (EDL) is almost doubled in PV-/- mice [4]. Mitochondrial proteins are differently affected in TA by PV deficiency. Cytochrome c oxidase subunits I (COX1) and Vb (COX5b), as well as cytochrome c, are significantly upregulated, while ATP synthase subunit β shows only a minor increase [5]. Besides ATP production, mitochondria are crucial for cellular Ca^2+^ signaling. They are dynamic structures as their relative density and intracellular distribution, morphology and physiology vary in different cells and tissues. The organelle composition is adapted to meet the metabolic and signaling needs of each cell [6]. The inverse correlation of PV expression levels and mitochondria content observed in fast-twitch muscles is also found in PV-ergic neurons. In PV-/- Purkinje cells, the relative mitochondrial mass in the soma is augmented by 40% [7], while ectopic expression of PV in neurons considerably decreases the mitochondrial volume (by almost 50%) evidenced in striatal neurons [8]. Mechanisms implicated in the inverse regulation were investigated in a gain-of-function model (PV-negative C2C12 cells as controls and C2C12 clones stably expressing PV) and a loss-of-function model, i.e. in TA from PV-/- and wildtype mice [9]. The inverse, bidirectional regulation of mitochondrial volume and PV expression was corroborated however, in two separate models. Analysis of pathways implicated in this regulation revealed an involvement of the PGC-1α/SIRT1 signaling axis.

Besides PV’s expression in excitable cells, it is also expressed in epithelial cells lining certain tubules in distinct regions of the distal nephron of the kidney. In humans and mice, PV is expressed exclusively in the early part of the distal convoluted tubule (early DCT/DCT1). In rats however, PV expression is observed in the thick ascending limb of the loop of Henle, the late DCT, connecting tubules (CNT) and in intercalated cells of the collecting duct [2,10]. The distal nephron plays an important role in the reabsorption of NaCl and in the fine-tuning of the final excretion of Ca^2+^, Na^+^ and Mg^2+^ [11]. In the mouse, the major sites of active, transcellular Ca^2+^ transport are DCT2 and, probably to a lesser extent, CNT. Ca^2+^ enters the cell at the luminal membrane via the TRPV5 channel and is sequestered by the intracellular “Ca^2+^ shuttles” calbindin D-28k (CB-D28k) or calbindin D-9k (CB-D9k). At the basolateral side, Ca^2+^ ions are extruded into the blood via the Na^+^/Ca^2+^ exchanger NCX1 and the plasma membrane Ca^2+^-ATPase PMCA1b [12,13]. The early DCT is also considered to be the major site of active transcellular Mg^2+^ reabsorption. Mg^2+^ enters the cell through apical TRPM6 channels [14], while the nature of the putative intracellular Mg^2+^ shuttle and the extruder protein at the basolateral side has not been disclosed yet. TRPM6, the gatekeeper of transcellular Mg^2+^ reabsorption, is co-expressed with PV. As PV contains so-called Ca^2+^/Mg^2+^ mixed sites, PV is assumed to play a role not only in Ca^2+^-buffering/shuttling, but also in the regulation of Mg^2+^ homeostasis. In line, PV expression levels are upregulated in mice subjected to dietary Mg^2+^ restriction [15,16].

PV-/- mice exhibit a reduced expression of the thiazide-sensitive sodium-chloride co-transporter (NCC) in the early DCT in the absence of ultrastructural changes. They show increased diuresis and kaliuresis at baseline, with higher aldosterone levels and lower lithium clearance [17]. Of note Mg^2+^ levels in the serum and in the urine are normal; PV-/- mice do not waste Ca^2+^, they even have a positive Ca^2+^ balance with increased bone mineral density [17]. This phenotype is reminiscent of the manifestations of Gitelman’s syndrome, an inherited salt-losing tubulopathy with loss-of-function mutations in the NCC co-transporter.

Here we used a transgenic mouse line expressing the enhanced green fluorescent protein (EGFP) under the control of the PV promoter crossed with PV-/- mice [18,19]. These mice express EGFP in the “PV-ergic” cells allowing for their identification, however not expressing PV protein. As PV is localized in the early DCT, large-particle based flow cytometry technique (COPAS) facilitated the selective isolation of DCTs from PV+/+ and PV-/- mice. The aim of this study was to gain insight into the regulation mechanisms of the Ca^2+^ homeostasome in non-excitable, PV-expressing cells. The inverse regulation of PV and mitochondria demonstrated before in muscle cells and Purkinje cells was validated in DCT cells of the kidney. DCT cells without PV manifest an increase in the expression of the mitochondrial marker COX1. The mechanism functions likewise in an *in vitro* cell culture system, i.e. in Madin-Darby canine kidney (MDCK) cells. Ectopic expression of PV decreased COX1, silencing of PV in the same PV-overexpressing cells via *Pvalb* shRNA restored the initial situation. The relative mitochondrial volume in PV-positive MDCK cells was strongly reduced and the mitochondrial membrane potential collapsed at lower CCCP concentration compared to PV-negative control MDCK cells. Thus, the previously observed inverse regulation of mitochondria and PV in excitable cells seems to be a general hallmark also prevailing in non-excitable cells of epithelial origin. The PV-induced reduction in mitochondrial volume and changes in the mitochondrial protein composition is proposed to impair the mitochondria’s ability to uphold its membrane potential, when the cytosolic Ca^2+^ concentration is augmented.

## Materials and Methods

### Transgenic mice

The transgenic mouse line B6,D2F2Tg^(Pvalb-EGFP)1Hmon^ (kind gift of H. Monyer, Heidelberg) expresses EGFP under the control of the PV (mouse gene symbol: *Pvalb*) promoter thus allowing to identify PV-ergic cells [20]. The EGFP expression pattern corresponds well to the PV distribution in the kidney [2]. PV-EGFP PV-/- mice were generated as described before [18] by crossing PV-EGFP mice with the constitutive knockout mice for PV (B6.Pvalb^tm1Swal^; [3]). In the resulting line, EGFP is expressed in the population of “PV-immunoreactive” cells, e.g. in PV-ergic neurons in absence of PV expression [18]. Mice were kept on a standard diet with free access to food and water under a day/night cycle of 12/12 hours. All animal experiments in this study were performed in accordance with the guidelines of the European Committee Council Directive of November 24, 1986 (86/609/EEC) and the study was approved by the Veterinary Office of the Canton of Fribourg, Switzerland under the authorization: 2013_13_FR. Kidney removal was performed under ketamine/xylazine anesthesia and mice were sacrificed under anesthesia by transcardial exsanguination.

### Isolation of distal convoluted tubules using Complex Object Parametric Analyzer and Sorter (COPAS) sorting

For the COPAS sorting, we used the protocol described by Markadieu et al. [21]. Briefly, 8-weeks old mice were anesthetized by a mixture of ketamine/xylazine and perfused transcardially with ice-cold Krebs buffer. The kidneys were removed, the cortex was minced and incubated in Krebs buffer (pH 7.3) containing collagenase type 1 (1 mg/ml; Sigma, Buchs, Switzerland) and hyaluronidase (2000 U/ml; Sigma, Buchs, Switzerland). The tubule suspension was filtered through a 190-μm and a 100-μm sieve and finally collected on a 40-μm sieve. Tubules were then sorted by the COPAS methodology (Union Biometrica, Holliston MA). EGFP-positive (EGFP^+^) tubules mostly consisting of DCTs [15] were collected in 1.5 ml Eppendorf tubes coated with 0.5% BSA (800 tubules per tube). Tubes were placed on ice, the tubules finally pelleted by centrifugation (800 x g, 5 min, 4°C). From these DCTs total RNA and proteins were extracted.

### Real-time quantitative PCR

Total RNA of DCT segments (1600 DCT tubules) was extracted with peqGOLD TriFast^™^ (Peqlab, Erlangen, Germany) following the manufacturer’s protocol. Contaminating DNA was removed by incubation with DNase. cDNA was synthesized with ImProm-II™ Reverse Transcriptase (Promega, Dübendorf, Switzerland). The resulting cDNA was diluted 1:4 prior to use. PCR amplicons from the cDNA (in triplicates) were generated on a Rotor-Gene 6000 thermocycler (Corbett Research, Mortlake, Australia) using LightCycler^®^ 480 SYBR Green I Mastermix (Roche Diagnostics, Mannheim, Germany), a one-component hot start reaction mix for PCR containing FastStart Taq DNA Polymerase and DNA double-strand-specific SYBR Green I dye for product detection and characterization. The primer pairs all spanning an exon-exon junction are listed in Table 1 and were individually added to the master mix. After an initial denaturation step (95°C, 10 min), 40 cycles were run (denaturation: 95°C, 15 s; annealing: 60°C, 30 s; elongation: 72°C, 20 s). The absence of nonspecific byproducts or primer dimers was verified by a melting curve (60–95°C).

**Table 1:** Primers used for genotyping and qRT-PCR.

Protein /Abbreviation	Gene Symbol	5’ primer	3’ primer
uncoupling protein 2	Ucp2	TGGTAGCCACCGGCAGCTTT	ACGGGGACCTTCAATCGGCAA
EF-hand domain-containing protein D1; mitocalcin	Efhd1	TGGACATCCACCAGGGTACAGCG	AGCCGTCCCTTCCAGCATCATAC
mitochondrial calcium uptake 1	Micu1	AAGCAGCCAGAACACTTGGGCC	AGCCCACACTCTCCAAGGCTGTA
mitochondrial calcium uniporter	Mcu	CTCACCAGATGGCGTTCGAGTCG	GCGTCGCTGCATCTTCATGGCT
mitochondrial calcium uniporter regulator 1	Mcur1	ACGCTCTGGTGTGCTTACTT	AGAGCGATTTCCTGCTGCAT
cytochrome c oxidase subunit 1 (COX1)	COX1	TACCCACCTCTAGCCGGAAA	ATGGCTGGGGGTTTCATGTT
ATP synthase beta subunit	Atp5b	ACCTCGGTGCAGGCTATCTA	AATAGCCCGGGACAACACAG
ribosomal protein S23	Rps23	ATGCCTTGTGGGTCCTTCCTGC	ACGACACTTGCCCATCTTGCCGG
ubiquitin C	Ubc	CGGAGTCGCCCGAGGTCACA	CTGCATCGTCTCTCTCACGGAGTT
parvalbumin (PV)	Pvalb	AGTCGTTTAAACATGTCGATGACAGACTTGCTC	AGTCACTAGTTTAGCTTTCGGCCACCAGAGT

### Quantitative Western blot analysis

For Western blot analyses, the lysate of 800 DCTs or 20–40 μg protein extract (MDCK cells) were loaded and separated on polyacrylamide gels (10% for COX1 and ATP synthase subunit β; 15% for PV), then transferred onto nitrocellulose membranes using a semidry transfer protocol. After blocking with 5% solution of non-fat milk in TBS buffer, membranes were incubated with the relevant primary antibodies (α-PV25; Swant, Marly, Switzerland 1:5,000; α-COX1, Invitrogen, Zug, Switzerland 1:2,000; α-ATP synthase subunit β, Invitrogen 1:5,000). Horseradish peroxidase-coupled secondary antibodies (Sigma, Buchs, Switzerland) were used at a dilution of 1:10,000. The protein bands were visualized with the Immobilon Western AP Substrate (Millipore, Zug, Switzerland). Imaging and analysis of the blots were performed with the FluorChem E system (Cell Biosciences, Santa Clara, USA). Membranes were stained with Ponceau S to check the loading and to enable normalization for densitometry. The integral of all visible protein bands was used in order to prevent a bias from any single reference or housekeeping protein [22,23].

### Immunohistochemistry

Kidneys were fixed with 4% PFA and frozen in liquid nitrogen. Cryosections (5 μm) were mounted on microscope glass supports, blocked with 5% nonfat dry milk and then incubated 24 h at 4°C with α-PV25 (1:500; Swant). For the visualization, we used a fluorescent donkey anti-rabbit secondary antibody (1:500; Vector Laboratories, Burlingame, CA, USA). The incubation time was 2 h at room temperature. For the immunostaining of cells, sterile glass coverslips were placed in 6-well culture plates. MDCK cells were then grown directly on the coverslips, fixed with 4% PFA and stained with the antiserum against PV and the fluorescent secondary antibody. Pictures were taken with a Hamamatsu digital camera (C4742-95-12NR) mounted on an upright fluorescence microscope (Leica DM6000B, Heerbrugg, Switzerland). The data was processed using the Fiji (ImageJ) software.

### Cell culture

Madin-Darby canine kidney cells (MDCK) are derived from adult dog kidney and have properties similar to distal nephron cells [24]. They are widely used as a model cell line to study properties and regulation of distal renal epithelial cells. Cells were cultured at 37°C in a humidified atmosphere of 95% air and 5% CO_2_ in Dulbecco’s modified Eagle’s medium (DMEM high glucose) supplemented with 10% heat-inactivated fetal calf serum (FCS), 100 units/ml penicillin, and 100 μg/ml streptomycin. Cells were passaged by trypsinization every 2–3 days.

### Stable gene transfer

pLVTHM-PV lentiviral vector (Addgene plasmid #12247) was used to stably express parvalbumin in MDCK cells. The lentiviral construct and the vector production were described by [25]. Briefly, the GFP cassette in pLVTHM was replaced with rat *Pvalb* cDNA coding for full length PV. The cDNA fragment was synthesized by PCR using the primers 5’-AGTCGTTTAAACATGTCGATGACAGACTTGCTC-3’ and 5’-AGTCACTAGTTTAGCTTTCGGCCACCAGAGT-3’. The amplicon was digested with PmeI and SpeI and inserted into the PmeI and SpeI unique sites of the pLVTHM vector. Lentiviral vectors were produced by co-transfection of HEK293T cells with three types of plasmids: the transfer vector pLVTHM, the envelope vector pMD2.G-VSVG and the packaging vector pCMVDR8.91 (in a ratio of 10:3:8). Lentiviral vectors in the supernatant of HEK293T cells were harvested 48 and 72 h after transfection. MDCK cells were seeded in 24-well plates. The following day, they were transfected with non-concentrated lentivirus suspension containing 8 μg/ml Polybrene (Sigma, Buchs, Switzerland). Clones were selected by serial dilutions, expanded and tested for PV expression by Western blot analysis using the PV25 antibody.

### Down-regulation of PV expression in MDCK cells using lentiviral-mediated shRNA

PV was downregulated in MDCK cells by short-hairpin RNA (shRNA). Plasmids coding for shRNA against rat *Pvalb* (pLKO.1-Pvalb) were purchased from Sigma, Buchs, Switzerland. Lentiviral vectors were produced as described above. For the transduction, MDCK cells were seeded into 6-well plates (50,000 cells per well) and grown for 24 h. Lentivirus containing *Pvalb* shRNA was added at a MOI of 10 and 20. After 3 days of incubation, the medium was removed and replaced with growth medium containing 2 μg/ml puromycin. Cells were kept for 10 days in the selection medium; total proteins were then extracted with RIPA buffer and analyzed by Western blot.

### Measurement of the mitochondrial membrane potential (ΔΨ_m_)

For the detection of mitochondrial membrane potential changes two different fluorescent probes were used: Mitotracker Red (MTR)/Green (MTG) (Life Technologies, Luzern, Switzerland) and JC-1 (Enzo Life Sciences, Lausen, Switzerland). Cells were seeded in 96-well plates (10,000 cells/well; 8 wells/treatment) and incubated for 24 h at 37°C. The dyes were prepared in culture medium at a final concentration of 50 nM (MTR), 100 nM (MTG) and 5 μM (JC-1). To dissipate the mitochondrial membrane potential, various concentrations of carbonyl cyanide m-chlorophenyl hydrazone (CCCP) were used. Cells were incubated for 45 min in the dye solution containing increasing amounts of CCCP (37°C), then washed with PBS to remove the free dye. The fluorescence was measured in a Victor^™^ X3 2030 multilabel reader (Perkin Elmer, Schwerzenbach, Switzerland).

### Flow Cytometry

To assess the cellular mitochondrial mass, cells were stained with MTG-FM (200 nM), a mitochondrial-specific fluorescent dye showing a distinct fluorescence independent of the mitochondrial membrane potential. Control cells and PV-expressing MDCK cells were trypsinized, normal growth medium was added to stop the reaction. After centrifugation (1000 x g, 3 min), cells were resuspended in PBS and counted. The cells were stained for 30 min at 37°C. After the incubation, the cells were washed and resuspended in PBS at a concentration of 1x10^6^ cells per ml. Cell counts were performed on a BD Accuri C6 flow cytometer (BD Biosciences, Allschwil, Switzerland). The forward scatter *vs*. side scatter of untreated cells was used to determine the size gating. Quantitative and statistical analyses were performed with the FlowJo software (Tree Star, Inc., Ashland, OR, USA).

### ATP measurements in MDCK cells with or without PV expression

Relative ATP levels were determined using the ATP Bioluminescence Assay Kit CLS II (Roche) according to the manufacturer’s instructions. Briefly, 9 volumes of boiling 100 mM Tris/4 mM EDTA (pH 7.75) were added to one volume of the cell suspension. After 2 min of incubation at 100°C, the samples were centrifuged at 1000 x g for 60 s, the supernatant was then transferred to a fresh tube. Samples (50 μl) were pipetted into a 96-well plate and 50 μl luciferase reagent were added; the luminescence was measured in a Victor^™^ X3 2030 multilabel reader (Perkin Elmer, Schwerzenbach, Switzerland). Bioluminescence signals were normalized to total protein contents (Coo Protein Assay, Uptima, Interchim, Montluçon, France) in the samples, the latter reflecting the number of cells in each well. Results are from 4 independent experiments, each one carried out in triplicates. In each experiment the value for control MDCK cells (without PV expression) was defined as 100%.^®^

## Results

### Absence of PV alters the transcriptome of PV-expressing DCT segments

**Fig 1:**
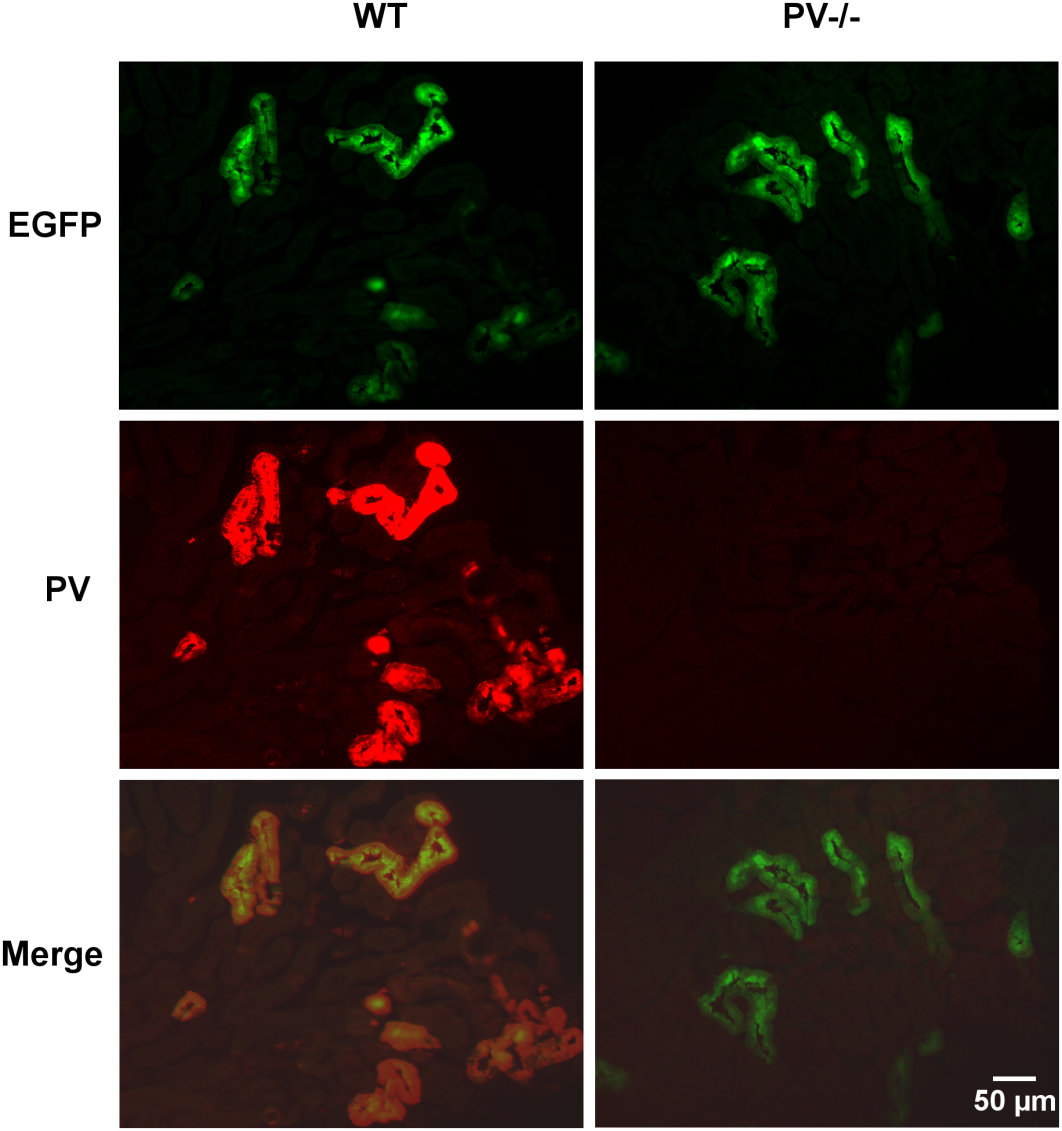
Immunohistochemical staining for PV (red) and EGFP (green) in the kidney of wildtype (WT) or PV-deficient (PV-/-) mice. In WT (PV-EGFP) kidney, the expression of EGFP (upper panel) and PV (middle panel) is restricted to DCT1 and shows 100% colocalization (lower panel). The qualitative distribution of EGFP is the same also in EGFP-PV PV-/- mice, however endogenous PV is completely absent.

**Fig 2:**
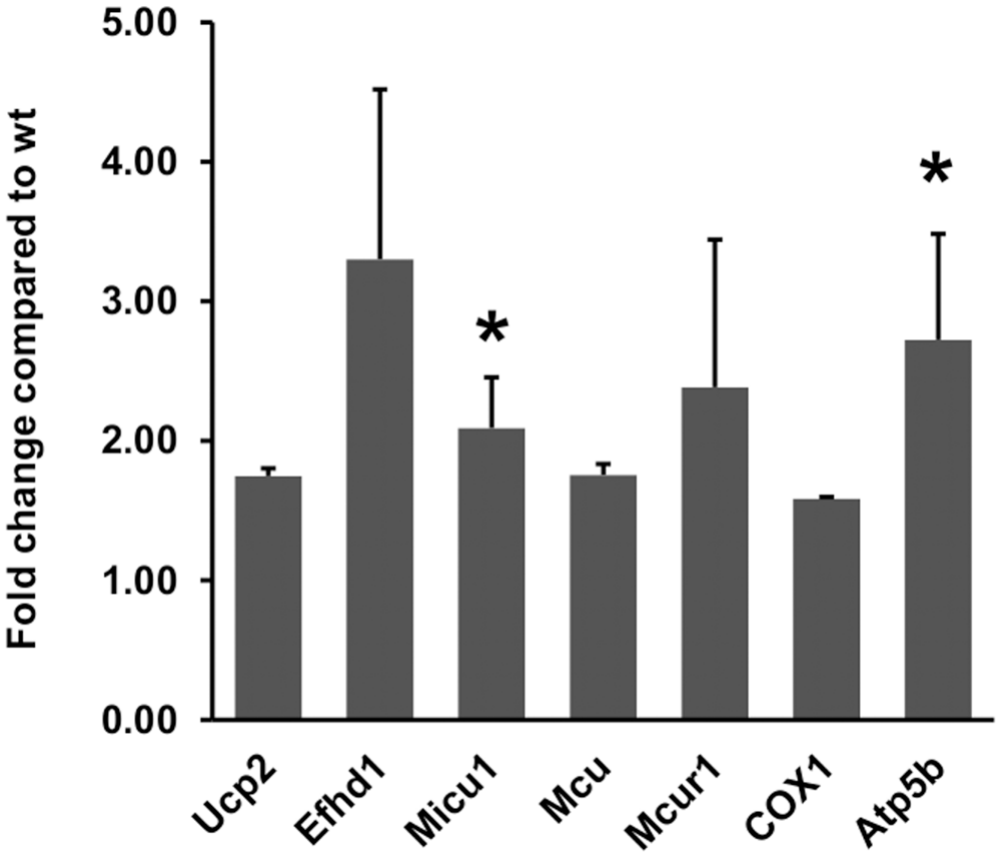
Relative mRNA expression levels of 7 selected genes determined by qRT-PCR based on results from Gene Chip Analysis (for more details, see S1 Table). RNA levels of genes implicated in mitochondrial Ca^2+^ regulation: *Efhd1*, *Micu1*, *Mcu*, *Mcur1* as well as other mitochondrial genes (*Ucp2*, *COX1* and *Atp5b*) are upregulated in PV-deficient DCT cells (results are from n = 4 animals per genotype, mean ± sem. The upregulation is statistically significant for *Micu1* and *Atp5b* (p<0.05, Mann-Whitney U test).

**Fig 3:**
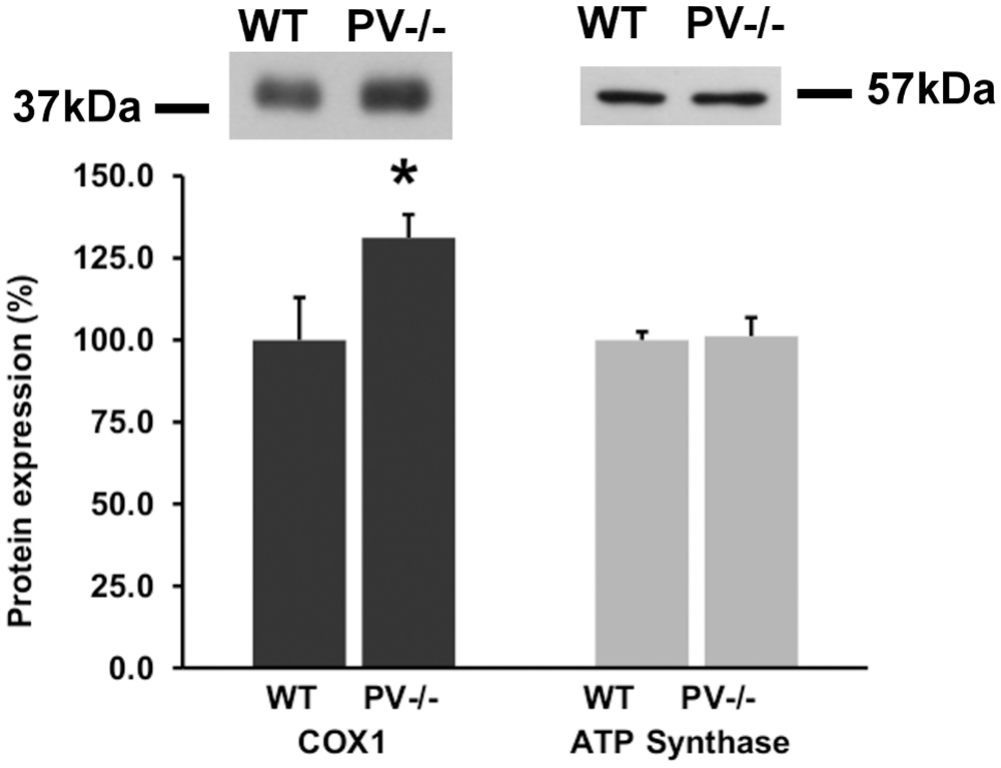
Western blots for COX1 and ATP synthase subunit β in DCT lysates from 4 control (WT) and 4 PV-/- mice. Values are mean ± sem of 3 independent experiments. In PV-/- mice the expression of COX1 was augmented by 31.2 ± 7.0% compared to WT (*p = 0.03706), while no changes were observed for ATP synthase (n.s.). Representative Western blot signals are shown in the upper part.

DCT segments from mice expressing EGFP under the control of the *Pvalb* promoter [20], either control mice with PV expression (PV+/+) or PV-deficient mice (PV-/-) were isolated using a Complex Object Parametric Analyzer and Sorter (COPAS), an established method to isolate specific tubular fragments of the nephron [15,21,26]. Beforehand, in order to verify that EGFP-expressing tubule segments are identical to segments with endogenous PV expression, kidney sections from EGFP-PV+/+ mice were stained for either EGFP or PV (Fig 1); the merged images demonstrated essentially 100% overlap. The EGFP expression pattern was the same in EGFP-PV-/- mice, but evidently no specific PV signal was observed (Fig 1). RNA from both genotypes (3 mice per group) was extracted and used for Affymetrix Gene Chip Analysis. Initial analyzes revealed a vast number of signals to be significantly up- or downregulated in DCT of PV-/- mice (S1 Table). As previously reported for fast-twitch muscle and PV-overexpressing myotubes [5,9], signals for several genes coding for mitochondrial proteins (*Ucp2*, *Efhd1*, *Micu1*, *Mcu*, *Mcur1*, *COX1*, *Atp5b*) were increased. In order to validate the Gene Chip data, total RNA was extracted from a second set of COPAS-isolated DCT fragments of 4 PV+/+ and 4 PV-/- animals and mRNA levels of seven genes coding for mitochondrial proteins were determined by quantitative RT-PCR. Besides prototypical mitochondrial genes including cytochrome c oxidase subunit 1 (*COX1*), ATP synthase subunit β (*Atp5b*), uncoupling protein 2 (*Ucp2*), we focused on genes implicated in mitochondrial Ca^2+^ handling: mitocalcin (*Efhd1*), mitochondrial calcium uptake 1 (*Micu1*), mitochondrial calcium uniporter (*Mcu*) and mitochondrial calcium uniporter regulator 1 (*Mcur1*). In comparison to PV+/+ mice, mRNA levels in PV-/- DCT were upregulated for all investigated genes (Fig 2). The same set of genes was also analyzed in TA of mice with or without PV expression. All seven genes were also upregulated in TA at the level of mRNA (S1 Fig). With respect to protein expression, the mitochondrial encoded cytochrome oxidase c subunit 1 (COX1), part of complex IV that participates in the transport of protons across the inner mitochondrial membrane and in establishing the mitochondrial membrane potential (**Δ**Ψ_m_), was increased by 31% ± 7% in the DCT of PV-/- mice (Fig 3), while protein levels of ATP synthase subunit β were unchanged. Thus, results from qRT-PCR and Western blot analyses suggested that the absence of the cytoplasmic Ca^2+^ buffer PV altered the mitochondrial protein composition: up-regulating proteins implicated in Ca^2+^ handling and generation of **Δ**Ψ_m_, likely without increasing ATP production evidenced by unchanged ATP synthase subunit β and also by increased *Ucp2* mRNA levels. Thus, we postulated that in the absence of PV, mitochondria are modified to better uphold **Δ**Ψ_m_ necessary to drive mitochondrial Ca^2+^ uptake, thus emphasizing the role of mitochondria as temporary Ca^2+^ stores and involved in the shaping of Ca^2+^ transients [27]. To further gain insight in mechanisms of PV and mitochondria regulation, we used a model system, i.e. MDCK cells representing dog kidney distal nephron cells [24].

### Inverse regulation of PV and mitochondria in MDCK cells

**Fig 4:**
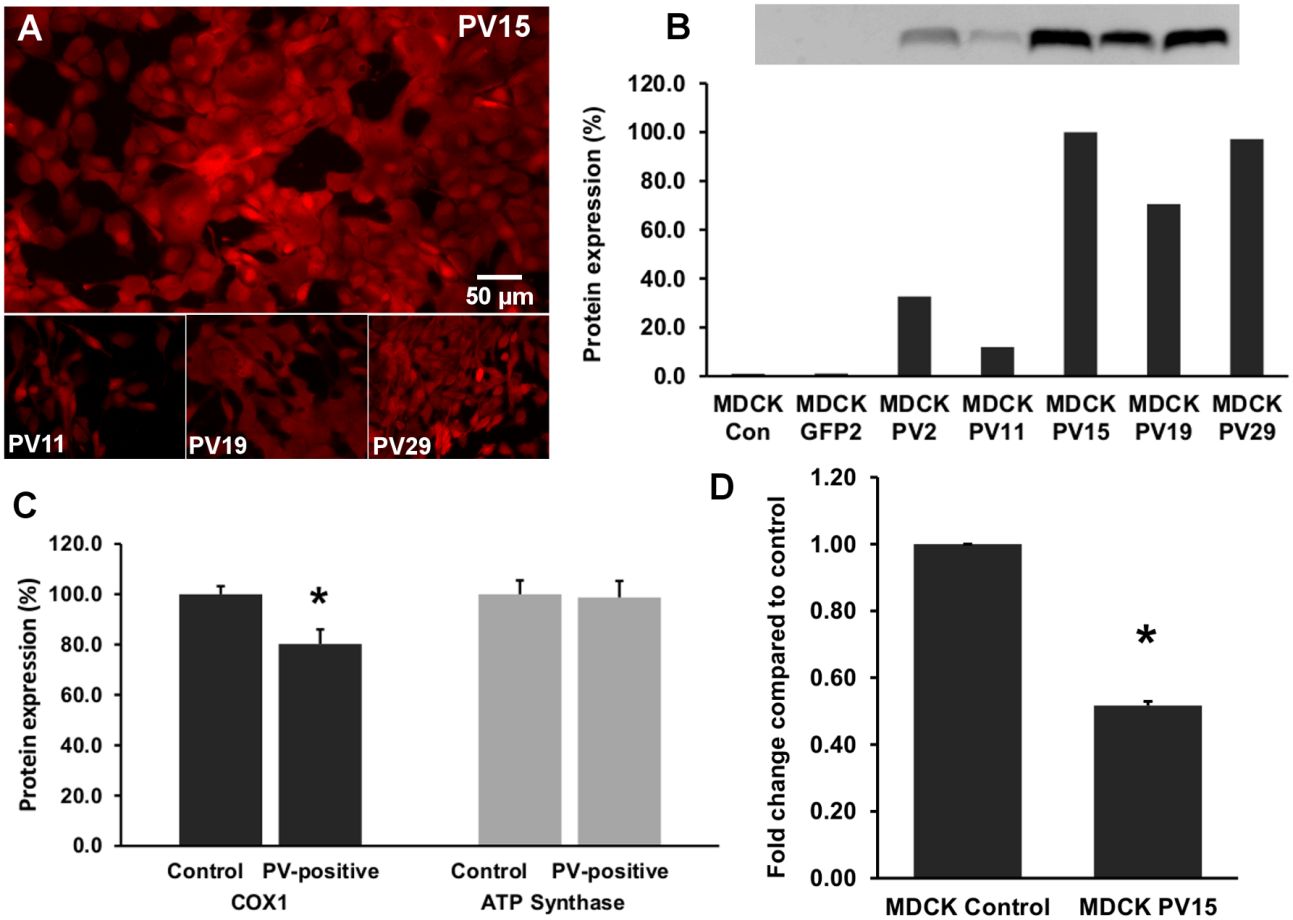
Madin-Darby canine kidney (MDCK) cells stably expressing PV. Lentiviral infection of MDCK cells led to cytoplasmic expression of PV evidenced by PV immunohistochemistry; PV expression levels were variable in clones selected by serial dilutions (A). Relative PV protein expression levels of selected clones were quantified by Western blot analyses (B). Non-transfected (Con) and EGFP-transfected clones (EGFP2) were negative for PV. Expression levels in individual clones were considerably different; e.g. levels in clone PV11 were almost 10-fold lower than in the high-expressing clone PV15. COX1 and ATP synthase subunit β protein levels of clones without PV and clones highly expressing PV (PV15, PV19, PV29) were compared (C). COX1 was significantly reduced in the PV-expressing clones (*p = 0.0099), ATP synthase subunit β levels were not different; p = 0.890823; n = 6 independent experiments, mean ± sem). The mRNA level of *COX1* gene coding for COX1 was assessed by qRT-PCR (D). In the PV-expressing PV15 clone, the *COX1* signal was decreased by 48%; p = 0.00005). The results are the mean of 2 independent experiments (mean ± sem).

**Fig 5:**
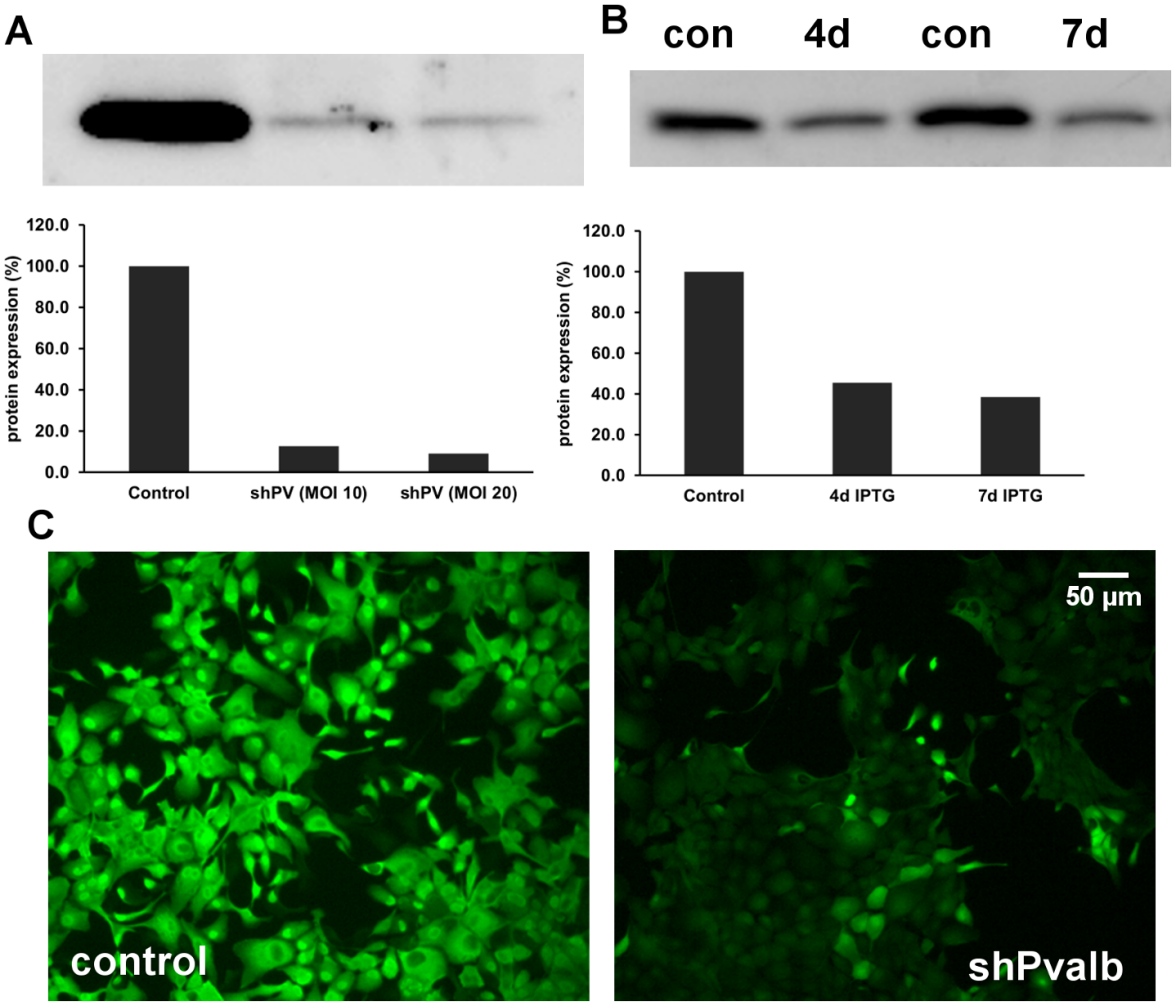
Silencing of ectopic PV in MDCK cells (clone PV15). A) Down-regulation of *Pvalb* mRNA via constitutive lentiviral-mediated shRNA decreases PV protein expression levels after 10 days of puromycin selection. B) PV down-regulation is less potent via the IPTG-inducible shRNA MISSION^®^ system tested in the PV-positive MDCK clone PV15. C) IHC of PV clone PV15 infected with constitutive lentiviral shPvalb after 10 days of puromycin selection (right image) in comparison to untreated cells (left image).

**Fig 6:**
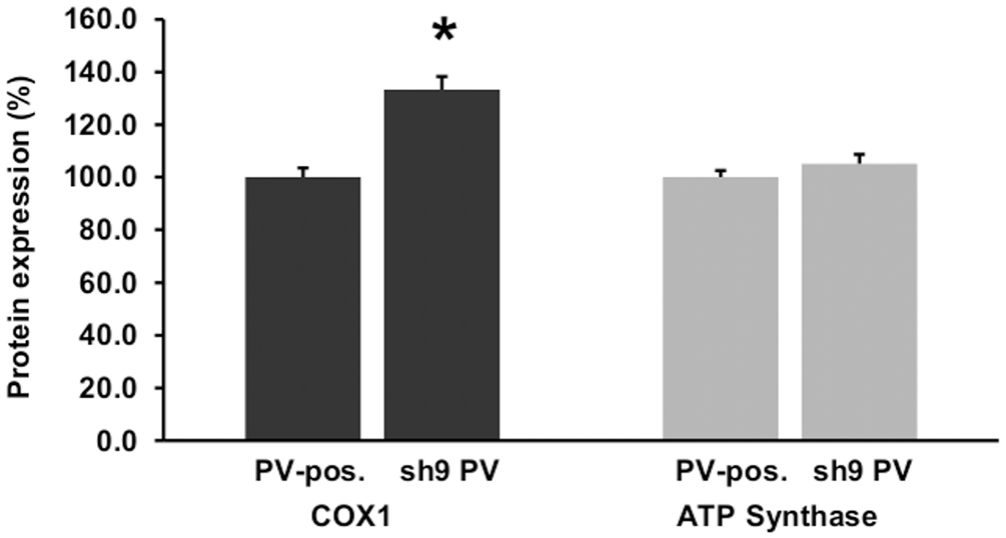
Western blot of COX1 and ATP synthase subunit β protein expression after PV silencing via shPvalb. Down-regulation of PV induced a significant rise in COX1 expression (*p = 0.00002, n = 4 independent experiments, mean ± sem), ATP synthase subunit β expression was unchanged (n = 3 independent experiments, mean ± sem; n.s.).

In fast-twitch muscle and cerebellar Purkinje cells exemplifying excitable cells, an inverse regulation of PV and mitochondria was demonstrated previously [7–9]. However, whether such a regulation also occurs in non-excitable cells, and whether it operates bi-directionally was yet unknown. In initial Western blot experiments we ascertained that no other major Ca^2+^-binding proteins, acting as putative cytosolic Ca^2+^ buffers were present in MDCK cells; results for PV, CB D-28k and CB D-9k were all negative. MDCK cells were infected with lentivirus (pLVTHM-PV vector; [25]) stably expressing PV in MDCK cells. From the total of PV-expressing MDCK cell clones characterized by variable expression levels, individual clones were selected by serial dilution, expanded and then screened for PV expression by IHC (Fig 4A). PV expression levels of clones were determined semi-quantitatively by Western blot analysis; non-infected MDCK cells and pLVTHM-EGFP infected MDCK cells served as negative controls (Fig 4B). In order to compare relative PV expression levels, the signal of clone PV15 having the highest PV expression level was defined as 100%. High PV levels were detected in clones PV15, PV19, PV29, clearly lower ones in clones PV2 and PV11 (Fig 4B). No PV signal was detected in control MDCK and in EGFP-lentivirus infected MDCK cells. Since we expected the largest differences to exist between control and high PV-expressing MDCK clones, quantitative expression levels of COX1 and ATP synthase subunit β were determined in the high PV-expression clones PV15, PV19 and PV29 and compared to control MDCK cells. The mean COX1 level of the 3 PV-clones was significantly reduced, while ATP synthase subunit β levels were not affected by the overexpression of PV (Fig 4C). With respect to COX1, this is the inverse of what was observed in DCT of PV-/-mice. qRT-PCR of RNA isolated from clone PV15 revealed the *COX1* mRNA coding for COX1 to be down-regulated (Fig 4D). In order to demonstrate the reversibility of the effect, PV expression in pLVTHM PV-infected clone PV15, was silenced by two different approaches: constitutive down-regulation of PV via lentiviral-mediated shRNA and IPTG-inducible down-regulation using the MISSION^®^ Inducible shRNA system. The constitutive method reduced PV expression levels to about 10% of the initial amount evidenced after 10 days of puromycin selection (Fig 5A), while with the inducible system, PV expression levels decreased to 45% after 4 days of IPTG treatment and to less than 40% after 7 days of IPTG treatment (Fig 5B). IHC for PV using a fluorescent secondary antibody confirmed the strong silencing effect of the constitutive lentiviral-mediated shRNA (Fig 5C). As the result of the PV-down-regulation was more robust with the constitutive shRNA expression system, this one was chosen for the determination of COX1 and ATP synthase subunit β levels by Western blot analyses. Lentiviral shRNA-silencing of ectopically expressed PV in MDCK cells led to a significant increase in COX1 compared to the untreated PV-positive cell clone PV15 (Fig 6). Also the mitochondrial volume determined by FACS analysis was increased after *Pvalb* mRNA silencing, from 65.8 ± 11.5% to 79.2 ± 14.1% (p<0.05). Thus, the changes induced by ectopic PV expression were reverted partially by *Pvalb* shRNA treatment documenting the bidirectional regulation in an identical setting. The partial reversal of the mitochondrial volume might be the result of the incomplete PV down-regulation by the shRNA approach. ATP synthase subunit β levels were unchanged after PV down-regulation (Fig 6), i.e. neither PV’s up- nor its subsequent down-regulation affects ATP synthase subunit β levels. These experiments clearly demonstrated that PV and COX1 were bi-directionally and inversely regulated in MDCK cells.

### Control MDCK cell mitochondria are better suited to withstand CCCP-induced membrane depolarization than PV-expressing MDCK cells

**Fig 7:**
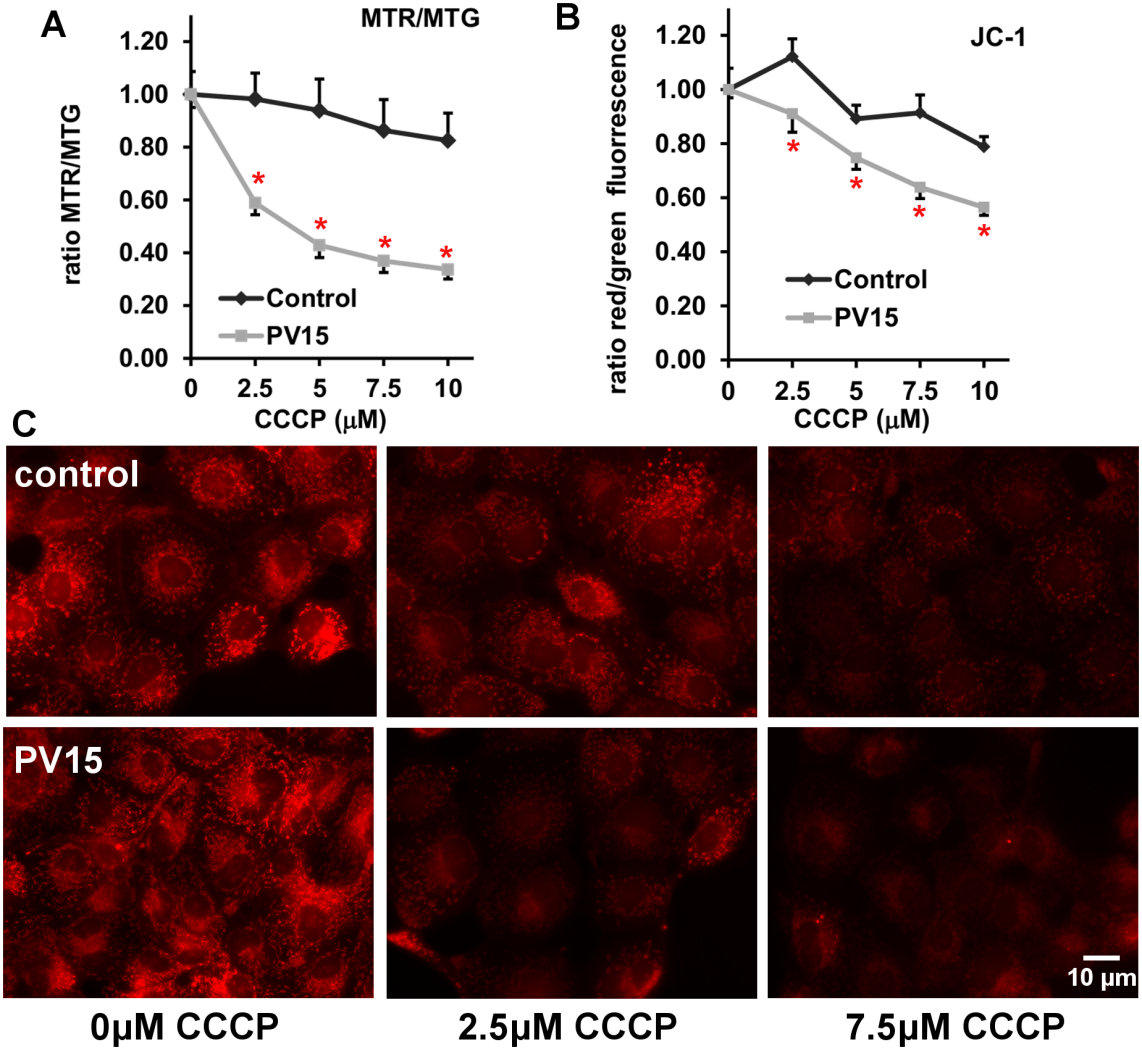
CCCP-induced changes in the mitochondrial membrane potential evaluated by two fluorescent lipophilic cationic dyes: Mitotracker Red (MTR)/Green (MTG) (A) and JC-1 (B). Treatment with the uncoupler CCCP decreased the mitochondrial membrane potential. The concentration dependent decrease was bigger in the PV-expressing MDCK PV15 cells compared to control MDCK cells (n = 3 independent experiments, mean ± sem, differences between control and PV15 were statistically significant at all CCCP concentrations (pairwise t-test; p<0.05 marked with (*) for all pairs in (A) and (B)). C) The cuvette measurements (A,B) were confirmed by qualitative fluorescence microscopy. Collapsing of the mitochondrial membrane potential by CCCP reduced MTR fluorescence emission in both control (upper row) and PV15 MDCK (lower row) cells; the decrease was more pronounced in the PV-expressing PV15 clone.

The precise mitochondrial protein composition with respect to expression levels of individual protein is assumed to be the result of tissue- or even cell-specific mitochondria function [6,28]. Based on this assumption, we hypothesized that mitochondria from PV-expressing and non-PV-expressing MDCK cells would differ in their capacity to withstand Ca^2+^ influx-induced membrane depolarization. For this we relied on fluorescent dyes suitable to measure changes in the mitochondrial membrane potential. Since intracellular fluorescence increases as the result of either a rise in **Δ**Ψ_m_ or an increase in mitochondrial mass density, a method of controlling for changes in mitochondrial mass is required to monitor changes in **Δ**Ψ_m_ [29]. We used two approaches, either the ratiometric dye (JC-1) or a combination of two dyes, Mitotracker Red (MTR) sensitive to **Δ**Ψ_m_ and Mitotracker Green (MTG) that is insensitive to changes in **Δ**Ψ_m_. Normalized ratios for both approaches demonstrated that an increase in the concentration of the uncoupler CCCP in the range of 2.5–10 μM led to a minor decrease in **Δ**Ψ_m_ in control cells (Fig 7A and 7B). In PV-expressing PV15 cells, CCCP induced a strong, concentration-dependent decrease of the red/green fluorescence ratio (Fig 7A) and JC-1 (Fig 7B) fluorescence. Representative images of MTR (red) fluorescence in MDCK cells without (control) or with PV expression (clone PV15) are depicted in Fig 7C. Qualitative staining was similar in control and PV15 MDCK cells in the absence of the uncoupler. The collapsing of the mitochondrial membrane potential by addition of CCCP caused a loss in red fluorescence in mitochondria, at 2.5 μM mostly in PV15 cells (Fig 7C). At the higher CCCP concentration (7.5 μM), mitochondrial staining completely disappeared in PV15 cells, while in control MDCK cells, mitochondria staining was clearly reduced, but few visibly stained mitochondria were still present. The results from both approaches (JC-1 and MTR/MTG) demonstrate that the expression of PV impairs/attenuates the capacity of mitochondria to maintain **Δ**Ψ_m_ in the presence of CCCP. Next we investigated, whether these differences might be due, at least in part, to alterations in intracellular ATP levels in control MDCK and PV15 cells. Relative ATP levels were not different in PV15 and control MDCK cells maintained at basal cell culture conditions, i.e. during unperturbed cell proliferation *in vitro* (93.3 ± 12.1%, *vs*. 100 ± 3.9%, respectively; n.s., n = 4 independent experiments).

**Fig 8:**
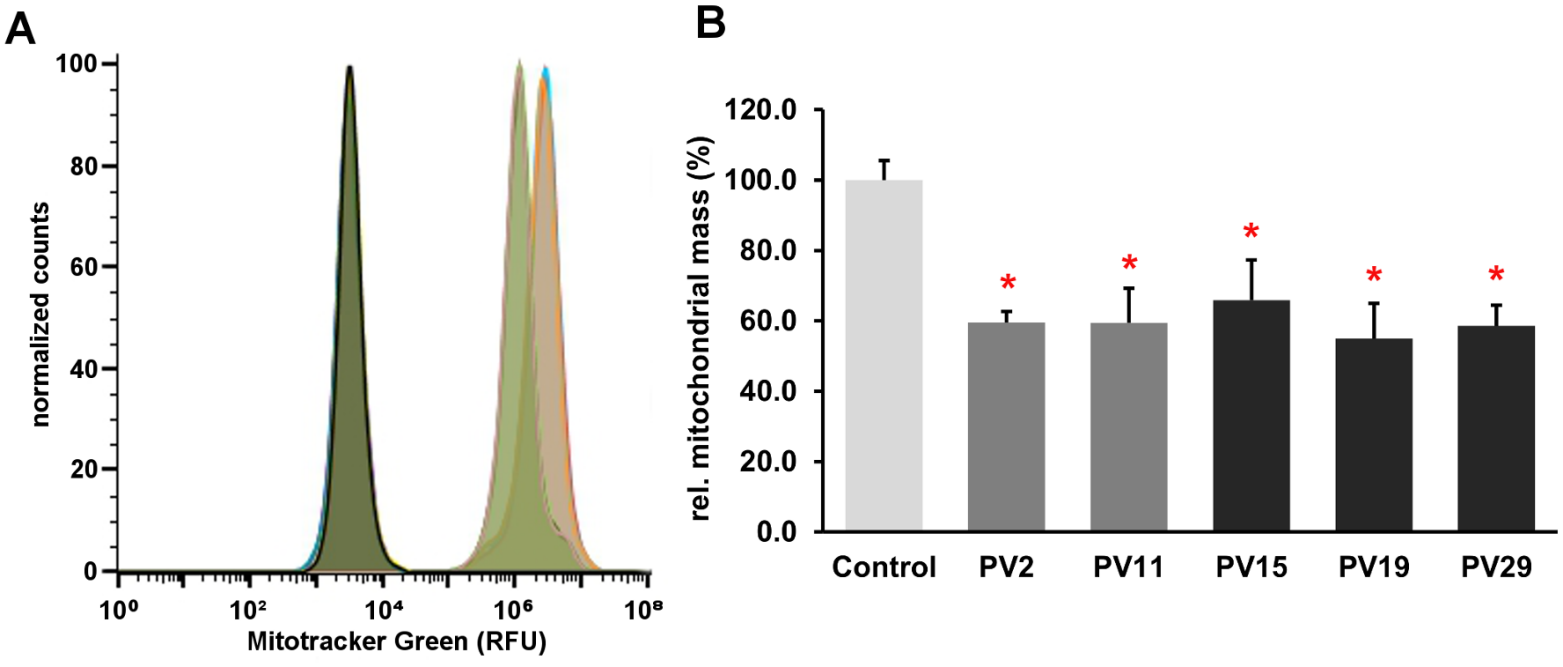
Evaluation of the relative mitochondrial volume in MDCK cells by FACS. A) The normalized histogram showed a left shift for the PV-positive MDCK PV15 (light green peak) compared to control MDCK cells (orange peak), the autofluorescence of the 2 cell lines was indistinguishable (overlapping dark green peaks). B) All PV-expressing MDCK clones exhibited a statistically significant reduction in the mean fluorescence intensity (n ≥ 2 experiments, mean ± sd). One-way ANOVA analysis resulted in a highly significant overall p-value (p = 7.23 x 10^−18^), indicative of differences between groups; within the group of PV-expressing clones no statistical differences were detected (p = 0.1589). A comparison of each PV clone with the control MDCK cells showed significant differences (t-test; p<0.05 for all PV clones).

Since an inverse regulation of PV and mitochondrial volume was demonstrated in excitable cells [4,7,8], we determined the relative mitochondrial volume in MDCK cells without or with PV expression. Mitochondria were stained with MTG, specifically accumulating in mitochondria regardless of the mitochondrial membrane potential; cells were assessed by FACS analysis. The normalized histogram of PV-positive PV15 cells showed a left shift, i.e. a shift to lower fluorescence intensities compared to PV-negative control cells (Fig 8A). The autofluorescence of unstained cells of both lines was the same. The mean of the fluorescence intensity was strongly reduced in PV15 cells (Fig 8B), likewise in all other MDCK clones stably expressing PV (Fig 8C). The reduction in mitochondrial volume was not linearly correlated with cytosolic PV expression levels. Clones with high PV expression (PV15, PV19, PV29; Fig 4) and clones with relatively low levels (PV2, PV11) showed the same extent of decrease in mitochondrial mass (Fig 8C). These results demonstrate that also in non-excitable epithelial kidney cells mitochondrial mass and the cytosolic Ca^2+^ buffer PV are inversely regulated. Thus, ectopic expression of the slow-onset Ca^2+^ buffer PV appears to functionally replace mitochondria conceivably based on similar kinetic properties of Ca^2+^ removal. The decrease in mitochondria volume together with the altered mitochondria protein composition impairs the epithelial cells’ ability to sequester Ca^2+^ into mitochondria. Putative consequences resulting from PV deregulation are discussed below.

## Discussion

Many physiological responses inside a cell and as well communication between cells are regulated by intracellular Ca^2+^ signals. Among these processes are transcription, cell cycle regulation, differentiation, cell motility/migration, programmed cell death, muscle contraction/relaxation, neurotransmission, secretion, and learning and memory [30]. To correctly process these seemingly simple signals, i.e. variations in the intracellular concentration of Ca^2+^, cells must be equipped with a precisely tunable system regulating the Ca^2+^ concentration accurately in space and time [1]. This Ca^2+^ signaling toolkit comprises Ca^2+^ channels, Ca^2+^ pumps and exchangers, as well as Ca^2+^ buffers. If a component of the toolkit is defective or missing, compensatory mechanisms are induced, either at the level of the affected cell or within the entire network, where the cell is functionally integrated. In general, the absence of a particular Ca^2+^-binding protein is not compensated by another EF-hand family member, i.e. one that is normally not expressed in this cell type [31]. In the subset of PV-ergic interneurons of PV-/- mice neither the distribution nor the levels of the Ca^2+^ binding proteins calretinin and CB-D28k were affected by the lack of PV [32]. Our experiments showed that ablation of PV in early DCT (DCT1) epithelial cells didn’t induce up-regulation of other cytosolic Ca^2+^ buffers in this part of the distal nephron. CB-D28k and CB-D9k, which are present in epithelial cells in close vicinity to the DCT1, exhibited unchanged expression levels and distribution patterns in PV-/- animals. The expression of CB-D28k commences abruptly at the transition early to late DCT and continues at a lower level throughout the connecting tubules. CB-D9k is expressed in late DCT and connecting tubules [2,10]. As demonstrated earlier in excitable cells, the loss of PV leads to an increased mitochondrial volume, assumed to represent an adaptive/compensatory mechanism. We used COX1 as a mitochondrial marker, since Racay et al. [5] had reported a rise in COX1 protein levels of 44% in TA of PV-/- mice (despite almost unchanged mRNA levels [5]), an effect similar to the one reported here in DCT of PV-/- mice (+31%). This indicates that similar compensatory (possibly homeostatic) mechanisms are present in excitable and non-excitable cells to compensate for the loss of PV. Of note, although the *Atp5b* mRNA coding for the ATP synthase subunit β was increased (Fig 2), no differences existed at the protein expression level (Fig 3). A similar situation prevails in TA of WT and PV-/- mice, and also in WT TA and slow-twitch muscle soleus (SOL), i.e. a minor increase in protein levels despite a larger increase in *Atp5b* mRNA [5]. Regulation of COX1, COX5b and ATP synthase subunit β in heart and liver is yet different from their regulation in muscle indicative of complex post-transcriptional control in different cell types, tissues [5] and likely also in different experimental conditions *in vitro* (this study).

The regulation of COX1 in MDCK cells functions bi-directionally: ectopic PV expression decreased COX1 by 20%, silencing of *Pvalb* mRNA by shRNA essentially restored the initial situation. Besides energy production, mitochondria are strongly implicated in controlling intracellular Ca^2+^ signaling and hence cell function by buffering of cytosolic Ca^2+^ levels [33]. The replacement of the cytosolic Ca^2+^ buffer PV by mitochondria might be seen as a homeostatic adaptation based on similar kinetic characteristics. PV is characterized as a slow-onset Ca^2+^ buffer in essentially all systems investigated. In muscle cells, PV is too slow to affect the rapid rise of the cytoplasmic Ca^2+^ concentration during the contraction phase, but it helps to increase the initial rate of decay of the transient and thus shortens the relaxation phase [31]. Uptake of Ca^2+^ into mitochondria follows changes in the cytoplasm with a slight delay. During a single twitch in TA recorded *in vivo*, mitochondrial Ca^2+^ signals reach their maximum with a delay of 10–20 ms compared to the cytoplasmic peak [34]. The mitochondrial Ca^2+^ peak is reached during the early relaxation phase. Thus, like PV, mitochondria promote an increase in the initial rate of cytoplasmic Ca^2+^ concentration decay. Most probably PV can’t be replaced by cytoplasmic Ca^2+^ buffers with fast kinetics. The absence of CB-D28k in Purkinje cells strongly affects the shape of dendritic Ca^2+^ signals [35] as well as the spine morphology [36]; however, it doesn’t increase mitochondrial volume density in PC somata [7]. Mitochondria up-regulation is essentially identical in PV−/− and PV/CB double knockout mice indicating that it is the absence of PV that induces the morphological upregulation of mitochondria [7].

PV expression and mitochondrial volume are inversely correlated [4,7–9], but whether this is causal and moreover whether it operates in both directions has not been investigated before. Like cytosolic Ca^2+^ buffers, mitochondria also have the ability to modulate cytosolic Ca^2+^ signals. This has been demonstrated in many cell types including MDCK cells [37]. Mitochondria have the capacity to take up Ca^2+^, store it transiently in the matrix and eventually release it back to the cytosol. These characteristics enable mitochondria to regulate cytoplasmic Ca^2+^ concentrations [38]. Mitochondria are highly dynamic structures, which adapt according to the specific demands in a cell. In addition to modifying the protein composition of the mitochondrial membrane, a volume increase/decrease might be another strategy to keep Ca^2+^ homeostasis under control. Gustavsson et al. [39] found a correlation of the mitochondrial mass and the timing of Ca^2+^ responses in pancreatic β cells. In addition they noticed that the Ca^2+^ rise was less pronounced in cell areas, where most mitochondria were localized. A buffering effect of mitochondria on cytosolic Ca^2+^ transients could also be confirmed in cardiomyocytes [40]. Down-regulation of MCU increased the peak amplitude of the cytoplasmic Ca^2+^ signal, but the decay rate tended to be lower. Peroxisome proliferator-activated receptor γ coactivator-1α (PGC-1α) is a regulator of mitochondrial biogenesis and modifies the mitochondrial proteome. PGC-1α overexpression in primary skeletal myotubes and in HeLa cells increased the mitochondrial volume [41]. Hence, intramitochondrial Ca^2+^ is distributed in a larger volume, resulting in a reduced increase of the mitochondrial Ca^2+^ concentration during mitochondrial Ca^2+^ accumulation. This reduced mitochondrial Ca^2+^ accumulation might lead to a diminished cellular sensitivity to Ca^2+^ -mediated apoptosis. Mitochondrial biogenesis is controlled by a Ca^2+^-regulated signaling pathway. Wu [42] analyzed the mitochondrial mass of myocytes of the plantaris muscle of transgenic mice expressing a constitutively active form of calcium/calmodulin-dependent protein kinase IV. Mitochondria of control cells occupied 24% of the total cross-sectional area of the myocyte, whereas the value for the transgenic was elevated to 38%. Likewise, the average thickness of the sub-sarcolemmal mitochondria layer was significantly higher. In our study, we measured the total mitochondrial mass of PV-expressing and PV-negative control MDCK cells by FACS analyses. MDCK cells strongly decreased the mitochondrial volume, when the cytoplasmic buffer PV was introduced. The correlation between mitochondrial volume decrease and PV expression levels is obviously nonlinear, since the decrease in volume was similar in clones with high and low PV-expression levels (compare Figs 4B and 8B). Thus, even low levels of PV appear sufficient to considerably decrease mitochondrial volume. The volume partially reverted towards the control situation, when PV expression in these cells was blocked by *Pvalb* shRNA. The reversal was not complete, likely resulting from the incomplete PV down-regulation after shRNA treatment as shown in Fig 5. Of note the decrease in COX1 protein expression in high PV-expressing clones (Fig 4C) and its reappearance after *Pvalb* shRNA treatment (Fig 6) was clearly more robust. This indicates that PV-induced changes in mitochondrial volume and COX1 expression levels are positively correlated, but not identically regulated by the presence or absence of PV. The integration of PV into the system modulating cytosolic Ca^2+^ transients reduces the need of mitochondrial Ca^2+^ buffering. As a consequence, the total mitochondrial volume was found to be decreased. Besides, the mitochondrial membrane composition is reverted to the actual demands of the cells. The relative levels of COX1 in ectopically PV-expressing MDCK cells were decreased.

Kidneys of PV -/- mice show a decreased expression of the thiazide-sensitive Na^+^-Cl^-^ co-transporter (NCC), colocalized with PV in the early DCT [17]. The absence of PV didn’t introduce any ultrastructural or apoptotic changes. NCC-/- mice on the other hand have drastically atrophied and shortened early DCTs, while the late DCT is intact [43]. Both mouse lines show a phenotype reminiscent of Gitelman’s syndrome, an autosomal recessive tubulopathy caused by inactivating mutations in the *SLC12A3* gene coding for NCC [44]. Gitelman’s syndrome is characterized by renal salt wasting, hypomagnesemia, hypokalemic alkalosis, hypocalciuria, and increased bone mineral density [45,46]. A link between NCC and PV was confirmed in mouse DCT (mDCT) cells. ATP (or UTP) promotes Ca^2+^ transients via luminal P2Y_2_ receptors and decreases the expression of NCC at mRNA and protein level. This Ca^2+^ signal was modulated by PV. NCC down-regulation was abolished in cells overexpressing cytosolic PV, but overexpression of nuclear-targeted or mutated PV unable to bind Ca^2+^ was not effective [17,47]. The PV-deficient mice used in our experiments displayed decreased NCC expression [17] in addition to the compensatory upregulation of mitochondrial volume. Replacement of cytosolic PV by mitochondria seems not adequate to fully control the amplitude (and possibly kinetics) of elevations of intracellular Ca^2+^ concentrations to stabilize NCC expression within the normal range as observed in cells from wildtype mice.

PV, a putative intracellular Ca^2+^ shuttle facilitating transcellular Ca^2+^ resorption, is not required for renal Ca^2+^ uptake. Mice lacking PV even have a positive Ca^2+^ balance: plasma Ca^2+^ levels are unchanged, calciuria is slightly reduced and bone mineral density increased. Hypocalciuria might be seen as a consequence of the NCC down-regulation, which leads to renal salt and water loss resulting in contraction of the extracellular volume (ECV). ECV triggers a compensatory increase in proximal Na^+^ reabsorption. This enhances the electrochemical gradient driving passive paracellular Ca^2+^ transport in the proximal tubule [48]. Additionally, extrarenal regulation mechanisms could be involved contributing to the hypocalciuria and the increased bone mineral density. In a Gitelman mouse model with NCC dysfunction [45], increased intestinal Ca^2+^ absorption and upregulation of the active Ca^2+^ transport machinery (TRPV6, CB-D9k, PMCA1b) were observed.

PV contains so-called Ca^2+^/Mg^2+^ mixed sites. It is therefore assumed that PV participates not only in Ca^2+^-buffering/shuttling, but also in the regulation of Mg^2+^ homeostasis. TRPM6, colocalized with PV in the early DCT, is considered to be the gatekeeper of Mg^2+^ reabsorption [11]. The activity of TRPM6 is tightly controlled by intracellular Mg^2+^. This implies that intracellular Mg^2+^ buffering and Mg^2+^ extrusion mechanisms strongly impact on the functioning of the channel [14]. Based on the affinity for Mg^2+^ (*K*_*D*_,_Mg_: _~_30 μM), PV is a suitable candidate to act as an intracellular transporter/shuttle [2]. PV-/- mice however have normal Mg^2+^ levels in blood/plasma and in urine. A possible explanation for these findings is that the lack of the Mg^2+^ shuttle PV is compensated by an increased passive paracellular uptake in the proximal tubules and the thick ascending limb of Henle or by intestinal hyperresorption of Mg^2+^. Alternatively it can’t be entirely excluded that PV is not contributing to the active Mg^2+^ reabsorption in the DCT. The molecular details of the Mg^2+^ extrusion across the basolateral membrane, as well as the Mg^2+^-binding molecules, are not known in detail yet. In a study of de Baaij et al. [15], the Mg^2+^-sensitive DCT transcriptome was elucidated; 46 genes showing differential expression were identified. *Pvalb* was among the upregulated genes, which hints at an involvement of PV in renal Mg^2+^ handling. In addition, yet unknown Mg^2+^-binding proteins might exist acting (possibly together with PV) as intracellular Mg^2+^ shuttles.

Our data show that the inverse regulation of PV and mitochondria is not restricted to excitable cells and moreover that it works bi-directionally. Also in kidney DCT1 epithelial cells, PV is replaced by mitochondria as evidenced by the increased mitochondrial volume. Our experiments further show that the mitochondrial membrane protein composition is adjusted to better preserve a high membrane potential and is optimally suited for mitochondrial Ca^2+^ uptake evidenced by the increase in proteins implicated in this process. Ectopic expression of PV induced the opposite changes that might be detrimental under some conditions.

## Supporting Information

S1 Fig qRT-PCR analysis of the same mitochondrial genes as shown in Fig 1. Total RNA was isolated from *tibialis anterior* of WT and PV-/- mice. All analyzed genes (mRNA) were increased in mice without PV expression. The increase was statistically significant (Mann-Whitney U test) for *Ucp2* (p = 0.042), *Micu1* (p = 0.022) and *Atp5b* (p = 0.026).
(TIF)

S1 Table Results from Gene Chip Analysis of selected mitochondrial genes. (DOCX)
