# Supplementary figures and images for: Antagonistic Regulation of Parvalbumin Expression and Mitochondrial Calcium Handling Capacity in Renal Epithelial Cells
Source: PLoS One. 2015 Nov 5;10(11):e0142005. doi: 10.1371/journal.pone.0142005 (PMC4634853; doi:10.1371/journal.pone.0142005)

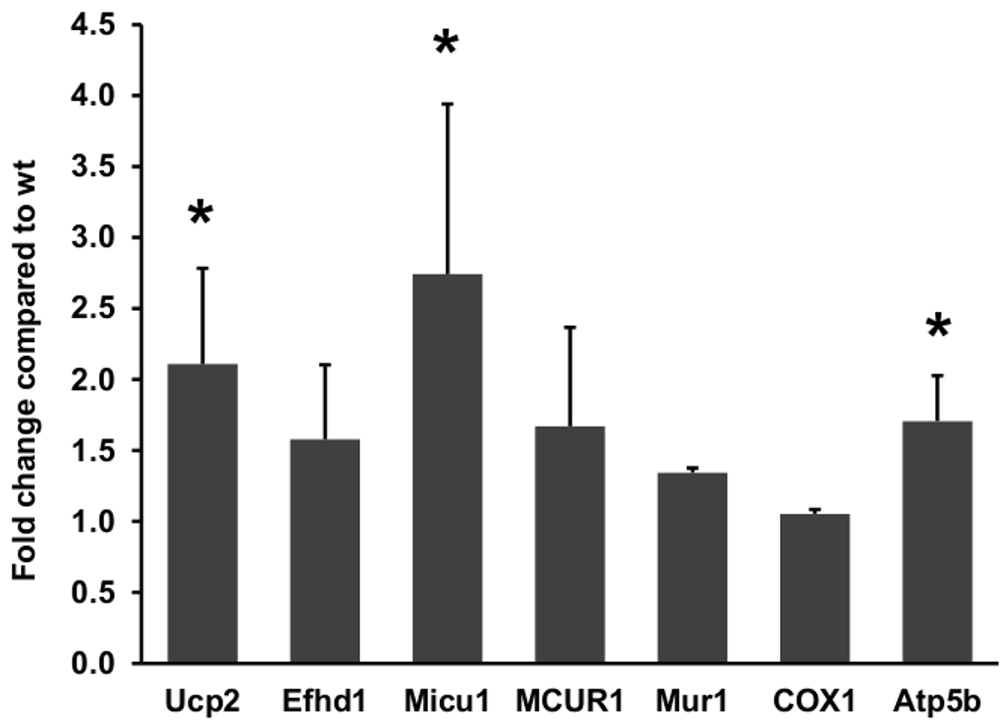

Supplement: S1 Fig — Total RNA was isolated from tibialis anterior of WT and PV-/- mice. All analyzed genes (mRNA) were increased in mice without PV expression. The increase was statistically significant (Mann-Whitney U test) for Ucp2 (p = 0.042), Micu1 (p = 0.022) and Atp5b (p = 0.026). (TIF) [file pone.0142005.s001.tif]
