# Supplementary material for: Antagonistic Regulation of Parvalbumin Expression and Mitochondrial Calcium Handling Capacity in Renal Epithelial Cells
Source: PLoS One. 2015 Nov 5;10(11):e0142005. doi: 10.1371/journal.pone.0142005 (PMC4634853; doi:10.1371/journal.pone.0142005)
Supplement: S1 Table — (DOCX) [file pone.0142005.s002.docx]

**S1 Table. Results from Gene Chip Analysis of selected mitochondrial genes.**

| **Protein /Abbreviation** | **Gene Symbol** | **Fold change** | **p-value** |
| --- | --- | --- | --- |
| uncoupling protein 2 | *Ucp2* | 1.649 | **0.000049** |
| EF-hand domain-containing protein D1; mitocalcin | *Efhd1* | 1.412 | 0.085 |
| mitochondrial calcium uptake 1 | *Micu1* | 1.068 | 0.418 |
| mitochondrial calcium uniporter | *Mcu* | 1.274 | **0.00027** |
| mitochondrial calcium uniporter regulator 1 | *Mcur1* | 1.279 | **0.026** |
| cytochrome c oxidase subunit 1 (COX1) | *COX1* | 1.056 | 0.254 |
| ATP synthase beta subunit | *Atp5b* | 1.091 | 0.126 |
